# Inflammatory biomarkers in axial spondyloarthritis and the effect of moderate and high-intensity interval training – a narrative review

**DOI:** 10.1007/s11033-025-11373-7

**Published:** 2025-12-19

**Authors:** Emma Lienesch, Johannes Voß, Åsa Andersson

**Affiliations:** 1 Halmstad University, Halmstad, Sweden; 2 Laboratory of Immunology, Institute of Nutritional Medicine, University of Lübeck and University Hospital Schleswig-Holstein, Lübeck, Germany; 3 Institute of Sports Medicine and Prevention, Leipzig University, Leipzig, Germany; 4 Dept. of Environmental- and Biosciences, FIH, Halmstad University, Halmstad, Sweden

**Keywords:** AxSpA, Biomarkers, Moderate-intensity training (MIT), High-intensity interval training (HIIT), Exercise, Physical activity

## Abstract

Rheumatic and musculoskeletal diseases pose a significant and increasing global burden, where axial spondyloarthritis (axSpA) shows a prevalence ranging between 0.2% and 1.6% worldwide. Alongside pharmacological treatments, non-pharmacological exercise interventions are a well-established option for axSpA, offering benefits such as improved disease activity, pain, mobility, and overall quality of life. Moderate-intensity training (MIT) is linked to anti-inflammatory effects, while high-intensity interval training (HIIT) shows promising results for cardiovascular health and to reduce systemic inflammation. Current research emphasizes the need to investigate the effects of MIT and HIIT on inflammation, bone remodeling, and tissue repair. Exercise influences inflammatory biomarkers like C-reactive Protein (CRP), Tumor Necrosis Factor-α (TNF-α), Interleukin-6 (IL-6), and calprotectin, though responses vary by intensity, duration, and disease stage. Further studies are crucial to optimize exercise regimens for axSpA patients by comparing moderate- and high-intensity training concerning their effects on selected blood biomarkers. The aim of this review is to summarize results from studies on specific biomarkers after exercise interventions in individuals with axSpA.

## Introduction

Axial spondyloarthritis (axSpA) represents a subset of inflammatory rheumatic conditions affecting the axial skeleton, primarily the sacroiliac joints and the spine, causing pain, fatigue, and stiffness [1–4]. Onset typically occurs before age 45, with prevalence rates varying between 0.3% and 1.4% across populations [4, 5]. The pathogenesis of axSpA involves chronic systemic inflammation [6] and pathological bone remodeling, including cartilage degradation and ossification [7]. Extra-musculoskeletal manifestations such as uveitis, psoriasis, enthesitis, spinal stiffness, and inflammatory symptoms are common, adding complexity to disease management [4].

Diagnosis often relies on the Assessment of SpondyloArthritis International Society (ASAS) classification criteria, based on X-ray findings, genetic markers like the HLA-B27 allele, and further clinical symptoms [4, 8]. Inflammatory biomarkers are important in predicting and monitoring disease progression and treatment responses. Serum proteins linked to inflammation, angiogenesis, and connective tissue turnover, such as cytokines, growth factors, and bone turnover markers, have been correlated with the disease progression and are suggested to serve as therapeutic targets [6, 9, 10].

The recommended management of axSpA by the ASAS and European League Against Rheumatism (EULAR) guidelines combines pharmacological and non-pharmacological treatment strategies [11, 12]. Pharmacological options include non-steroidal anti-inflammatory drugs (NSAIDs), as the conventional therapy, along with conventional synthetic (csDMARDS) and biological disease-modifying antirheumatic drugs (bDMARDs) such as tumor necrosis factor inhibitors (TNFi) and interleukin-17 inhibitors (IL-17i). While effective, these therapies are limited by potential unknown side effects, high costs, and accessibility challenges [13–15]. Non-pharmacological treatment options, particularly exercise, are emphasized for their ability to improve disease activity, spinal mobility, and the quality of life [13–17].

Moderate-intensity training (MIT) is beneficial for individuals with axSpA [11, 18]. However, high-intensity interval training (HIIT), which alternates between highly intense exercise and recovery periods, has, in addition, been shown to be effective in reducing cardiovascular risk in axSpA, where mortality rates are 20–40% higher in patients compared to the general population [19, 20]. Studies in other populations indicate that HIIT confers cardiovascular and systemic anti-inflammatory benefits [18, 21–23], yet its impact on biomarkers in axSpA remains unexplored. Given the potential therapeutic implications of exercise-induced control of systemic inflammation, a comprehensive review of existing evidence is warranted to clarify the relationship between exercise intensity and inflammation in axSpA, guiding future research and clinical applications. The comparison of the effects of MIT and HIIT on specific serum proteins commonly studied in axSpA is based on disease-specific biomarker concentrations. Current research highlights the influence of different training methods on marker concentrations following exercise.

The aim of this article is to investigate how different biomarkers are affected by exercise of varying intensity. These serum proteins play a role in inflammatory processes that contribute to the severity and progression of axSpA. For this purpose, literature searches were conducted in the PubMed, MEDLINE, SPORTDiscuss, CINAHL, and Google Scholar using the keywords “axial spondyloarthritis,” “biomarkers,” “exercise,” “moderate-intensity exercise,” and “high-intensity interval training,” while limiting the publication date from 1999 to 2024. Approximately 4855 articles were identified and 1551 were screened, of which 72 articles were ultimately included in the review.

## Moderate-intensity and high-intensity training in axSpA

AxSpA is a chronic inflammatory disease characterized by systemic inflammation, progressive structural changes, and increased cardiovascular risk. While pharmacological treatments such as TNF- and IL-17 inhibitors have significantly improved disease management, physical activity is a recommended and well-studied non-pharmacological treatment option for axSpA [13, 14]. However, there is no complete consensus about the most effective type, duration, and intensity of exercise to treat axSpA. Examining exercise-induced inflammation is particularly complex due to the interplay of numerous cells, inflammatory pathways, and mediators. Most research has focused on pro-inflammatory cytokines in athletes and in the general population, with limited attention to inflammation as a primary outcome in rheumatic musculoskeletal diseases [24]. The question therefore is, how training at different intensities affects various inflammatory proteins involved in the pathology behind axSpA.

### Moderate-intensity training

Regular, supervised moderate-intensity training (MIT), defined by reaching ventilatory and lactate threshold one during incremental tests [25], has shown significant benefits for axSpA patients, including improved disease activity, pain, spinal mobility, cardiorespiratory health, quality of life, and overall function [13–16]. Recommendations highlight cardiovascular, flexibility, and resistance training at moderate intensity for this population [11, 18]. MIT has been implicated to have anti-inflammatory properties, though studies focusing on changes in inflammatory biomarkers are limited [24, 26, 27].

### High-intensity interval training

HIIT enables repeated periods of exertion at or near maximum oxygen uptake, which are considered crucial stimuli for cardiorespiratory and metabolic adaptations to exercise [28]. Previous research on HIIT has focused on biomarkers in healthy adults, overweight individuals, and those with conditions such as metabolic syndrome, diabetes, or cardiovascular diseases with promising results to suggest a potential therapeutic relevance [21–23, 29]. In these cohorts, studies on the immediate effects of a single HIIT session suggest that changes in inflammatory biomarker levels depend on exercise intensity and individual´s metabolic state. Adipose tissue is a critical component in this context, acting as a metabolically active organ that regulates inflammation by releasing both pro- and anti-inflammatory cytokines [30, 31]. Recent findings indicate positive impact of HIIT on cardiovascular health, oxygen uptake, muscle metabolism, and systemic inflammation markers [18, 32]. Physical activity of higher intensity is suggested to provide increased cardiovascular protection in axSpA patients, compared to lower intensities [33, 34]. Concerns persist about high-intensity exercises exacerbating disease activity, given the elusive understanding of whether the beneficial effects of physical activity are local or systemic [35]. During vigorous muscle activity, myokines released affect various organ systems with an impact on metabolism, but also on inflammation. The impact of myokines extends to several physiological processes during extensive muscle engagement [36, 37], with muscle damage being of lesser significance in triggering inflammation [21, 23].

### Serum protein levels in axSpA and the effect of exercise at different levels

In musculoskeletal and chronic inflammatory conditions, biomarkers such as C-reactive Protein (CRP), Interleukin-6 (IL-6), and Tumor Necrosis Factor – alpha (TNF-α) are frequently studied as secondary outcomes, with regular physical activity linked to their reduction [26, 27] (Table 1). CRP is the most studied serum protein associated with axSpA [6] and commonly examined in clinical settings as an acute-phase protein reflecting systemic inflammation [3, 38]. TNF-α and IL-6, frequently used as biomarkers of inflammation, are known to stimulate CRP release from the liver [3, 26, 38]. The level correlates with structural damages on X-rays in early axSpA and the association of radiographic sacroiliitis [2]. However, CRP does not reflect the course of the disease [6] and has a low sensitivity and specificity, partly because only 40–50% of axSpA patients exhibit elevated levels [2]. The marker is also increased in individuals with non-specific lower back pain, those who are overweight, and is sex dependent [6, 39, 40]. It remains a dependable marker in the context of active axSpA and continues to be the most reliable biomarker associated with disease activity, predicting structural changes, and medication response [9, 20]. In addition to its role in axSpA, CRP is strongly associated with cardiovascular risk. A persistently elevated CRP level has been linked to increased cardiovascular mortality, independent of age, sex, and metabolic comorbidities, as well as a higher likelihood of experiencing fatal or non-fatal cardiovascular events [20]. Given its responsiveness to inflammation, anti-TNF-α therapy significantly reduces CRP levels, further supporting its role as a marker of both disease activity and therapeutic efficacy [38, 41, 42].

**Table 1:** Overview of selected inflammatory biomarkers and their role in AxSpA based on current literature

Biomarker	Role in axSpA	References
CRP	Included as an ASAS diagnostic criterion, CRP is only elevated in 40–50% of axSpA patients but remains the most reliable marker for disease activity, predicting structural changes and response to medication.	[2, 6, 8, 9, 20, 31, 51]
Calprotectin	Elevated serum calprotectin is a considered predictor of disease progression and syndesmophyte formation. It is hypothesized to reflect clinical improvement even in CRP-negative patients	[59–61]
TNF-α	Elevated concentrations are associated with restricted spinal mobility and indicate sustained synovial inflammation, making it a target for anti- TNF-α treatment.	[42, 45–47]
IL-6	Stimulates CRP production. IL-6 signaling is linked to severe progression in autoimmune diseases but is not exclusively disease-specific.	[6, 28, 38, 48, 68, 69]
MMP-3	Correlates with mSASSS, highlighting its role in bone formation processes and enabling the prediction of radiographic changes. It is significantly, though inconsistently, associated with diagnosis.	[6, 9, 38, 62, 63, 66]
VEGF	Suggested to be linked with the disease activity of axSpA, strongly elevated levels serve as precise predictors for radiographic spinal changes.	[6, 52, 53, 56]
HMW adiponectin	Inversely associated with radiographically visible spinal changes and in combination with VEGF and leptin a promising predictor of the disease course	[6, 45, 72]
Leptin	Inversely associated with radiographically visible spinal changes and in combination with VEGF and HMW adiponectin a promising predictor of disease course.	[6, 45, 72]

The role for MIT on serum inflammatory markers was studied by Isanejad et al. [43] in breast cancer survivors. Only a small, but non-significant, increase in IL-6 and TNF-α levels was observed. Together with CRP, TNF-α is most frequently investigated in association with chronic back pain [6], and target of the anti-TNF-α therapy used in axSpA [42]. TNF-α, a cytokine released primarily by macrophages during local and systemic inflammation, regulates the stimulation of various immune cells, affecting cell proliferation, differentiation, necrosis, or apoptosis [6, 44]. Elevated concentrations correlate with limited spinal mobility in axSpA patients [45]. In addition, TNF-α supports prolonged synovial inflammation by promoting ongoing IL-6 synthesis and by triggering the transcription factor NF-κB activation in fibroblast-like synoviocytes. TNF-α is known for suppressing osteoblast differentiation and is associated with bone destruction in inflammatory arthritis in general [46]. TNFi treatment effectively alleviates symptoms and improves function in axSpA patients with active disease, although variations in response remain unclear [47].

Exercise-induced reductions in TNF-a and IL-6 may require substantial fat loss, as adipose tissue contributes significantly to circulating levels of IL-6 [43]. Flynn et al. [26] also emphasized the role of adipose tissue and adiponectin in inflammation, highlighting the dual role of IL-6. IL-6, known as a pleiotropic cytokine, stimulates the production of CRP and is primarily secreted by diverse cell types, such as monocytes, fibroblasts, and endothelial cells [6, 38, 48]. Released in the acute phase of inflammation, in addition to being released in response to strenuous exercise, it participates in pro- and anti-inflammatory pathways, influencing metabolic control, bone metabolism, vascular health, and neural processes [6, 38, 48, 49]. Despite its role in inflammation, the clinical utility of IL-6 in axSpA remains debated. Unlike CRP, IL-6 is not disease-specific, as it is also elevated in individuals with non-specific back pain, with levels correlating more with pain intensity than disease activity [6]. However, some studies suggest IL-6 may complement other biomarkers in assessing disease severity and predicting treatment response [38]. Notably, anti-IL-6 therapies have been investigated in autoimmune diseases like rheumatoid arthritis, but their role in axSpA remains uncertain, with conflicting clinical trial results [47, 48]. Further research is needed to determine whether IL-6 inhibition could be a viable therapeutic strategy in axSpA. Exercise-induced IL-6 production is independent of TNF-α or NF-kB activation, alongside increased soluble TNF receptors, which naturally inhibit TNF-α and enhance anti-inflammatory effects during and after exercise [24, 50]. So, IL-6 acts as an anti-inflammatory molecule when released during muscle contractions but indicates disease or injury when driven by TNF-α [26].

Exercise-induced inflammation and myokine production from skeletal muscles benefit muscle hypertrophy, myogenesis, and systemic metabolic health [28, 36]. Studies examining the impact of MIT or HIIT on CRP and Vascular Endothelial Growth Factor (VEGF) concentrations are inconclusive due to varying disease status and group sizes [31, 51]. The serum protein VEGF is suggested to be associated with the disease activity of axSpA in combination with leptin and heavy-molecular-weight (HMW) adiponectin [6]. VEGF, a heparin-binding growth factor specific to vascular endothelial cells, stimulates angiogenesis by regulating proliferation and vascular permeability [52, 53]. Elevated serum levels of VEGF (above 600 pg/mL) serve as a precise predictor for radiographic spinal changes in axSpA patients, mainly in the case of an already increased risk due to syndesmophytes [53]. Secreted by various cell types, VEGF can trigger inflammatory cell activation and induces the production of pro-inflammatory mediators. Anti-inflammatory treatments, such as TNFi, have been shown to influence VEGF levels [54, 55]. In response to exercise, VEGF promotes capillarization in skeletal muscle tissues [56]. VEGF is one of the central signaling factors in angiogenesis, regulating the proliferation of endothelial cells and vascular permeability [52, 53]. Prior research in healthy adults has not shown any short-term effects of HIIT specifically on VEGF levels [57, 58].

Calprotectin has been linked to a dual impact on health, providing protective effects against certain cancers while also potentially contributing to immune dysfunction following intense physical exertion [59]. During muscle contractions, skeletal muscles generate calprotectin mRNA, leading to a rise in plasma levels immediately after aerobic exercise, which can persist for at least one hour in healthy individuals [60]. This increase is influenced by both the intensity and duration of exercise, with the highest levels observed after prolonged and vigorous activity [59]. Interestingly, research by Levitova et al. [60] demonstrated that intensive exercise resulted in a marked decrease in serum calprotectin levels among axSpA patients, which was associated with improvements in disease activity and spinal mobility. Calprotectin is a heterodimeric complex of two calcium-binding proteins, S100A8 and S100A9, that functions as an acute-phase reactant during inflammation [59, 61]. Primarily stored in the cytosol of granulocytes, it has both extracellular and intracellular functions. When released, calprotectin acts as a damage-associated molecular pattern, intensifying inflammatory responses [61]. In axSpA, monocytes infiltrate inflamed synovium and produce calprotectin, identified in the synovial tissue of axSpA patients [9, 60]. The serum calprotectin level correlates with disease progression, syndesmophyte formation, and intestinal involvement, especially when combined with CRP. Calprotectin reflects clinical improvement even in CRP-negative patients, making it a potentially more reliable marker of disease activity. Unlike CRP, calprotectin levels appear unaffected by NSAIDs or bDMARDs, suggesting that it may provide a more stable reflection of underlying inflammation regardless of treatment status [60]. This characteristic, combined with its association with structural progression, underscores calprotectin’s potential as a complementary or superior biomarker to CRP in axSpA.

Matrix Metalloproteinase 3 (MMP-3) facilitates the migration of satellite cells through the basal lamina [62]. Satellite cells contribute to muscle hypertrophy by inserting their nuclei into growing muscle fibers. There is evidence suggesting that metalloproteinases, particularly MMP-3, are involved in the inflammatory responses [6]. Matrix metalloproteinases are calcium-dependent and zinc-containing endopeptidases. Their function is to degrade extracellular matrix proteins, thus contributing significantly to cellular processes [6]. MMP-3, produced in response to pro-inflammatory cytokines, is the metalloproteinase most studied in axSpA patients. It is significantly, but inconsistently, linked to the axSpA diagnosis [6, 63]. MMP-3 correlates with the modified Stoke Ankylosing Spondylitis Spinal Score (mSASSS), emphasizing the role of matrix metalloproteinases in bone formation processes [9, 38]. It also correlates with parameters of the ASAS classification criteria that reflect function, inflammation, and disease activity. This association enables to predict radiographic progression, especially in patients with pre-existing structural damage [9, 38, 64]. However, anti-TNF-α treatment significantly reduces serum MMP-3 levels, making it a valuable biomarker monitoring treatment response [63, 65]. Unlike CRP, which reflects systemic inflammation, MMP-3 may better represent local tissue inflammation and matrix degradation, providing additional insights into disease activity. However, further studies are needed to establish standardized MMP-3 thresholds for assessing treatment efficacy.

Urso et al. [66] found elevated MMP-3 concentrations following high-intensity training. Studies suggest that MMP-3 levels rise in response to mechanical stress induced by intense exercise [66]. This responsiveness induces adaptation and repair processes in joints, skeletal, and cardiac muscles [66] and mitigates exaggerated post-exercise inflammation [67]. A similar reaction is caused by IL-6, which triggers inflammation-limiting cytokines and suppresses TNF-α production [28, 68]. Studies have shown that young adults performing HIIT experience increased IL-6 and TNF-α concentrations in circulation shortly after exercise initiation [68, 69]. A pilot study supported the evidence of HIIT significantly influencing post-exercise IL-6 response in axSpA patients [70]. Despite its pro-inflammatory role, IL-6, released during muscle contraction, exhibits anti-inflammatory and endocrine functions [47], such as counteracting cardiovascular issues in rheumatic disorders [36].

### Clinical implications for exercise in axSpA and further research

In summary, intense and prolonged exercise generally leads to elevated inflammatory mediator levels, potentially increasing the risk of injury and chronic inflammation. Conversely, MIT or well-structured HIIT sessions with adequate recovery can provide significant anti-inflammatory benefits. Although cytokine responses to exercise vary by intensity and type, regular exercise demonstrates overall positive effects on inflammation markers [71]. This review summarizes current evidence on selected inflammatory biomarkers in axSpA and examines how moderate- and high-intensity exercise influences these markers, considering the elevated cardiovascular risks faced by axSpA patients due to systemic inflammation. HIIT may serve as a promising intervention to manage axSpA and associated conditions. Further studies are needed to clarify how inflammation is affected not only by the intensity of exercise, but also by its type and duration. Cerqueira et al. [71] highlighted these parameters as they may play a critical role in influencing inflammatory responses, particularly in individuals engaging in high-intensity exercise. Metsios et al. [24] recommended enhancing knowledge of how exercise impacts inflammation in rheumatic and musculoskeletal diseases. Future research of the role for exercise on disease parameters in axSpA needs to be carefully designed and adequately powered and should focus on examining how exercise influences inflammatory-, bone-, and tissue-remodeling markers. Furthermore, it is important to investigate the interplay between exercise and pharmacological treatments, including NSAIDs, corticosteroids, as well as conventional and biologic DMARDs, to provide a more detailed understanding of its role in managing inflammation within this population. Additionally, as Levitova et al. [60] proposed, patients with axSpA in the early stages of the disease may exhibit different responses to exercise compared to those with longer disease duration and varying levels of inflammation.

Given the complex interplay between inflammation, exercise, and disease progression, further research is needed to:

- Clarify the effects of exercise on inflammatory markers in axSpA, considering disease stage, intensity, and duration of training.
- Examine the interplay between exercise and pharmacological treatments, including NSAIDs, DMARDs, and biologics.
- Identify patient subgroups that may benefit the most from MIT or HIIT, particularly regarding cardiovascular risk management.

## Conclusion

Axial spondyloarthritis is a complex inflammatory disease requiring both pharmacological and non-pharmacological management. This review outlines the role of key biomarkers – such as CRP, IL-6, TNF-α, VEGF, MMP-3, and calprotectin – in inflammation, structural changes, and treatment response. While MIT consistently improves clinical outcomes, the effects of exercise on inflammatory biomarkers remain heterogeneous. HIIT has emerged as a promising intervention for improving cardiovascular health in axSpA, though concerns may persist regarding its potential to exacerbate inflammation.

Future research should clarify the dose-response relationship between exercise and inflammation, investigate synergistic effects with pharmacologic therapies, and identify patient subgroups most likely to benefit. Individualized exercise prescriptions, grounded in biomarker profiles and disease stage, may help optimize long-term outcomes in axSpA.

## Data Availability

No datasets were generated or analysed during the current study.
